# Correlating MRI features with additional genetic markers and patient survival in histological grade 2-3 IDH-mutant astrocytomas

**DOI:** 10.1007/s00234-023-03175-0

**Published:** 2023-06-15

**Authors:** Arian Lasocki, Michael E. Buckland, Tahlia Molinaro, Jing Xie, James R. Whittle, Heng Wei, Frank Gaillard

**Affiliations:** 1 Department of Cancer Imaging, Peter MacCallum Cancer Centre, Grattan St, Melbourne, Melbourne, Victoria 3000 Australia; 2 Sir Peter MacCallum Department of Oncology, The University of Melbourne, Parkville, Victoria Australia; 3 Department of Radiology, The University of Melbourne, Parkville, Victoria Australia; 4 Department of Neuropathology, Royal Prince Alfred Hospital, Camperdown, NSW Australia; 5 School of Medical Sciences, University of Sydney, Camperdown, NSW Australia; 6 Department of Medical Oncology, Peter MacCallum Cancer Centre, Melbourne, Victoria Australia; 7 Centre for Biostatistics and Clinical Trials, Peter MacCallum Cancer Centre, Melbourne, Victoria Australia; 8 Personalised Oncology Division, Walter and Eliza Hall Institute, Parkville, Victoria Australia; 9 Department of Medical Biology, The University of Melbourne, Parkville, Victoria Australia; 10 Department of Radiology, The Royal Melbourne Hospital, Parkville, Victoria Australia

**Keywords:** Radiogenomics, Imaging genomics, Glioma, Magnetic resonance imaging, Isocitrate dehydrogenase

## Abstract

**Purpose:**

The increasing importance of molecular markers for classification and prognostication of diffuse gliomas has prompted the use of imaging features to predict genotype (“radiogenomics”). CDKN2A/B homozygous deletion has only recently been added to the diagnostic paradigm for IDH[isocitrate dehydrogenase]-mutant astrocytomas; thus, associated radiogenomic literature is sparse. There is also little data on whether different IDH mutations are associated with different imaging appearances. Furthermore, given that molecular status is now generally obtained routinely, the additional prognostic value of radiogenomic features is less clear. This study correlated MRI features with CDKN2A/B status, IDH mutation type and survival in histological grade 2-3 IDH-mutant brain astrocytomas.

**Methods:**

Fifty-eight grade 2–3 IDH-mutant astrocytomas were identified, 50 with CDKN2A/B results. IDH mutations were stratified into IDH1-R132H and non-canonical mutations. Background and survival data were obtained. Two neuroradiologists independently assessed the following MRI features: T2-FLAIR mismatch (<25%, 25–50%, >50%), well-defined tumour margins, contrast-enhancement (absent, wispy, solid) and central necrosis.

**Results:**

8/50 tumours with CDKN2A/B results demonstrated homozygous deletion; slightly shorter survival was not significant (*p*=0.571). IDH1-R132H mutations were present in 50/58 (86%). No MRI features correlated with CDKN2A/B status or IDH mutation type. T2-FLAIR mismatch did not predict survival (*p*=0.977), but well-defined margins predicted longer survival (HR 0.36, *p*=0.008), while solid enhancement predicted shorter survival (HR 3.86, *p*=0.004). Both correlations remained significant on multivariate analysis.

**Conclusion:**

MRI features did not predict CDKN2A/B homozygous deletion, but provided additional positive and negative prognostic information which correlated more strongly with prognosis than CDKN2A/B status in our cohort.

## Introduction

Molecular markers have become critical to the classification of diffuse gliomas, being first integrated into the 2016 edition of the World Health Organization (WHO) Classification of Tumours of the Central Nervous System [1], and further increased in importance for diagnosis and prognosis in the recent 2021 edition [2] (henceforth “WHO 2021”). This has led to the emergence of the field of “radiogenomics” or “imaging genomics” — using imaging features to predict tumour genotype — which can address some of the challenges with attaining optimal genetic classification [3, 4]. For example, in grade 2–3 gliomas, the T2-FLAIR mismatch sign can predict an IDH [isocitrate dehydrogenase]-mutant astrocytoma without 1p/19q-codeletion (combined loss of the short arm of chromosome 1 and long arm of chromosome 19) with specificity close to 100% and high inter-observer agreement[5-10], albeit with a moderate sensitivity of about 42% on pooled analysis [8].

A potential criticism of the field of radiogenomics, however, is that molecular status is increasingly obtained as part of routine patient care, and the additional benefit of radiogenomics may be limited in this context [3]. Thus, it is important to determine whether MRI features such as the T2-FLAIR mismatch sign provide diagnostic and prognostic value beyond just prediction of genotype. This has potentially become more challenging with the release of WHO 2021, which added new molecular markers to the classification [2], and re-evaluation of existing knowledge is warranted. For example, contrast-enhancement — which is arguably the oldest MRI biomarker in diffuse gliomas — occurs both more commonly and to a greater degree in IDH-wildtype tumours [6, 10-16]. Thus, some of the negative prognostic implications of enhancement at initial diagnosis could potentially relate to this molecular association in and of itself.

Diffuse gliomas are stratified by IDH mutation status [2]. There are two types of IDH gene which are relevant to gliomas — IDH1 and IDH2 — with a mutation in either gene sufficient to consider the glioma IDH-mutant [17]. The IDH1-R132H mutation, which reflects replacement of arginine with histidine at codon 132 of the IDH1 gene, accounts for the majority of all IDH mutations [17]. This can be detected using routine immunohistochemistry, but is specific to the IDH1-R132H mutation and cannot detect other (non-R132H) IDH1 mutations or any IDH2 mutations (collectively grouped as “non-canonical” mutations). Given the importance of IDH status for stratification, for younger patients (under 55 years of age) with negative IDH immunohistochemistry, additional sequencing is recommended to potentially detect non-canonical mutations [1]. As all IDH1 and IDH2 mutations are considered equivalent for the purposes of diagnosis, they are generally combined in studies examining imaging features; thus, there is limited data [18] on whether the type of IDH mutation could influence the MRI appearances. This is clinically relevant, as it influences the likely value of IDH sequencing for a tumour which tests negative for an IDH1-R132H mutation but demonstrates imaging features suggestive of an IDH mutation, in particular T2-FLAIR mismatch.

An important refinement in WHO 2021 is the addition of homozygous deletion of CDKN2A and/or CDKN2B to the grading of IDH-mutant astrocytomas [2], related to the negative survival implications [19, 20]. Detection of homozygous CDKN2A/B deletion leads to classification as a grade 4 tumour, even in the absence of the classic grade 4 histologic features of necrosis or microvascular proliferation [2]. Given the recency of this change, the literature on correlating imaging features with CDKN2A/B status in gliomas is sparse [21, 22]. Furthermore, only a minority of such research has specifically examined imaging features associated with CDKN2A/B status in IDH-mutant astrocytomas that would otherwise be considered grades 2–3 [23], which is the group of gliomas for which CDKN2A/B is most relevant according to the 2021 classification [2].

The purpose of this study was to correlate conventional MRI features with homozygous deletion of CDKN2A and/or CDKN2B, type of IDH mutation (IDH1-R132H or non-canonical) and overall survival in a cohort of histological grade 2–3 IDH-mutant and 1p/19q-non-codeleted astrocytomas.

## Materials and methods

### Patient identification

Institutional Human Research Ethics Committee approval was obtained (HREC number QA2017093). Fifty-eight grade 2–3 IDH-mutant astrocytomas were identified retrospectively as part of a project correlating MRI features with genotype [24]. This comprised a consecutive cohort of patients diagnosed with a grade 2–3 glioma based on histologic criteria between September 2007 and December 2013, identified through the Central Nervous System Tumour Database at our hospital and previous research at our institution [7, 25]. Only patients with available pre-operative MRIs considered to be of diagnostic quality, including at least T2-weighted imaging, fluid-attenuated inversion recovery (FLAIR), and pre- and post-contrast T1-weighted imaging, were included. As a large tertiary referral centre, MRIs had been performed on a variety of scanners, including imaging incorporated from external institutions. As a result, imaging parameters varied.

All patients had next generation sequencing (NGS) [26] or IDH pyrosequencing to determine IDH status, unless an IDH1-R132H mutation had already been demonstrated by immunohistochemistry as part of routine clinical practice or previous research. IDH mutations were thus divided into IDH1-R132H (based on sequencing or positive immunohistochemistry) or non-canonical (all IDH1 and IDH2 mutations other than IDH1-R132H). 1p/19q status was determined by fluorescence in situ hybridisation, if previously performed as part of routine patient care, or by NGS if performed for the purposes of the study. NGS, which was performed in 50/58 patients, also included CDKN2A and CDKN2B deletion status.

### Background patient data

The following background data were obtained from patient records: age (taken at the time of initial surgical resection), sex, histological grade (grade 2 or 3), and date of death or last follow-up. Survival data were available for all but one of the patients. ECOG (Eastern Cooperative Oncology Group) performance status was obtained from the Central Nervous System Tumour Database and was available for all but six of the patients.

### MRI assessment

**Fig. 1:**
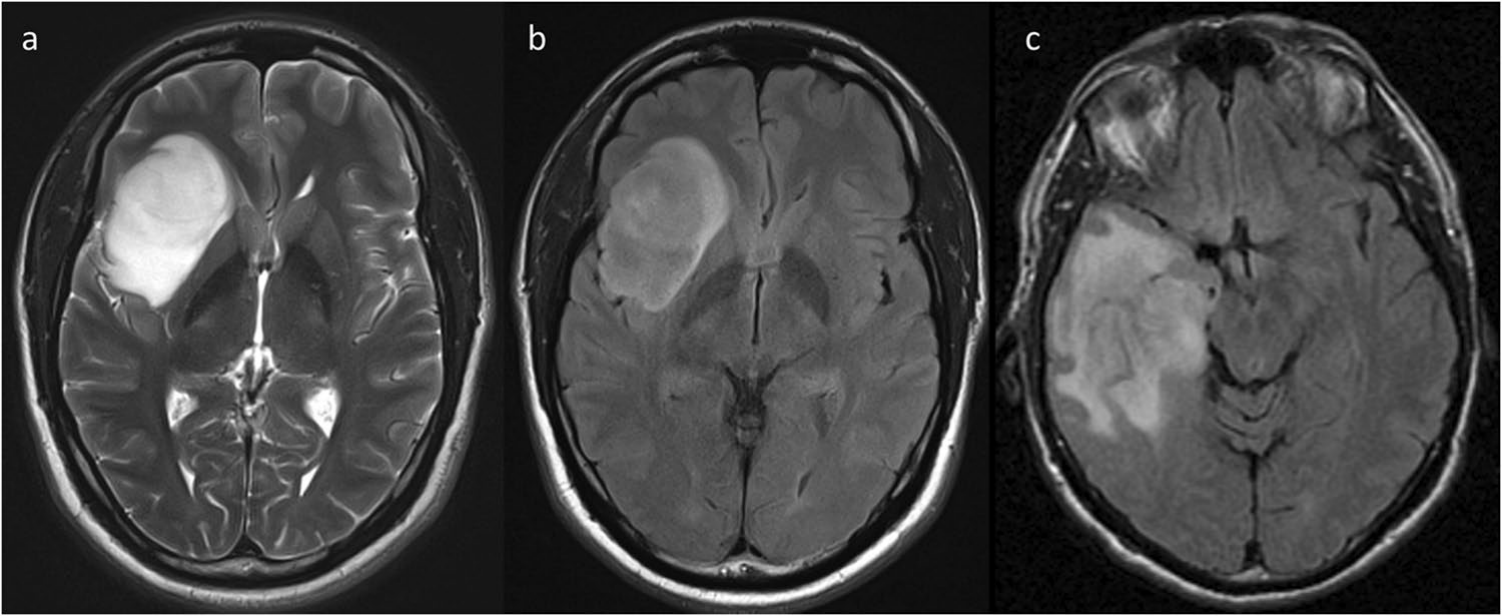
Representative patient images of the T2-FLAIR mismatch sign and tumour margins. The well-defined right fronto-insular tumour (**a** and **b**) demonstrates high T2 signal on T2-weighted imaging (left), with the majority of the lesion demonstrating substantially lower signal on FLAIR (middle), with a preserved FLAIR-hyperintense rim. In contrast, the right temporal tumour (**c**) in a different patient has ill-defined margins

**Fig. 2:**
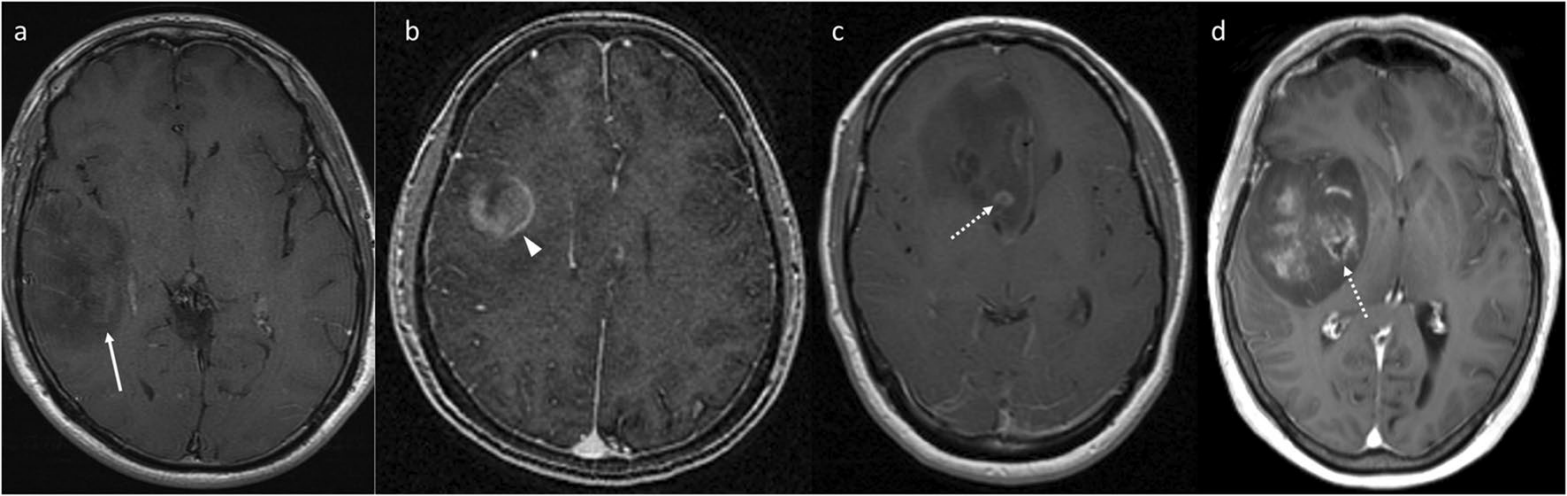
Representative images of enhancement and central necrosis in four different patients. The right temporal tumour (**a**) demonstrates wispy enhancement (solid arrow). The right frontal tumour (**b**) demonstrates solid enhancement (arrowhead); while there is a suggestion of a complete ring with central necrosis, the ring was considered incomplete anteriorly. In contrast, another right frontal tumour (**c**) demonstrates a small but complete enhancing ring (dotted arrow) with central necrosis, as does a right insular tumour (**d**)

Assessment was performed independently by two neuroradiologists (AL, FG) from different institutions with subspecialty expertise in neuro-oncology (having 8 and 13 years of experience), blinded to molecular status, histological grade (grade 2 or 3) and patient survival. The following MRI features were assessed, based on definitions provided in previously published research [24]: T2-FLAIR mismatch (stratified as <25%, 25–50% and >50%), well-defined tumour margins, the presence of contrast-enhancement (defined as absent, wispy or solid) and the presence of central necrosis. Of specific note, necrosis was distinguished from cystic change by being surrounded by a complete enhancing ring, and all tumours with evidence of necrosis were considered to be demonstrating solid contrast-enhancement. Representative patient images are provided in Figs. 1 and 2. Discrepancies were resolved by consensus. Good inter-observer agreement, with kappa above 0.6 for all of the included features, has already been reported for this cohort[24].

### Statistical analysis

Associations between MRI features and molecular features, including the presence of homozygous deletion of CDKN2A and/or CDKN2B, and type of IDH mutation (IDH1-R132H or non-canonical), were determined using the chi-square statistic. The Cox proportional hazard model was used to assess the associations between the overall survival and potential risk factors (demographic, molecular or MRI characteristics): age at surgery, sex, histological grade, ECOG performance status, type of IDH mutation, CDKN2A/B status, T2-FLAIR mismatch, well-defined tumour margins, enhancement and central necrosis. Factors with *p* <0.10 in the univariable Cox regression model were included in the multivariable Cox regression analysis, except for age and gender. Schoenfeld residual tests were used to evaluate the proportional hazard assumptions. Interactions between variables were analysed by adding interaction terms in the multivariable Cox regression model. This Cox regression model generates regression coefficients for the independent variables, the exponents of which reflect hazard ratios (HRs). Survival was assessed using Kaplan-Meier curves. A *p*-value less than 0.05 was considered statistically significant. All statistical analyses were performed in R (version 4.0.3) using standard and validated statistical procedures.

## Results

The mean patient age was 36.0 years, and 39 patients (67%) were male. Most tumours (51/58, 88%) were histological grade 2. The grade 3 tumours were associated were shorter survival (HR 3.09, 95% CI [confidence interval] 1.14–8.40, *p*=0.019), with a median survival of 6.0 years, compared to 10.0 years for grade 2 tumours. The majority of patients with available performance status data were ECOG 0 (46/52, 88%), but the minority with ECOG >0 (three ECOG 1, one ECOG 2 and two ECOG 3) had significantly shorter survival (HR 3.96, 95% CI 1.43–11.01, *p*=0.004). The majority of IDH mutations (50/58, 86%) were IDH1-R132H mutations, with the remainder (8/58, 14%) being non-canonical. Eight (16%) of the 50 tumours with available CDKN2A/B data demonstrated homozygous deletion; while these patients had slightly shorter survival (HR 1.37), the difference was not statistically significant (95% CI 0.41–2.08, *p*=0.571).

**Fig. 3:**
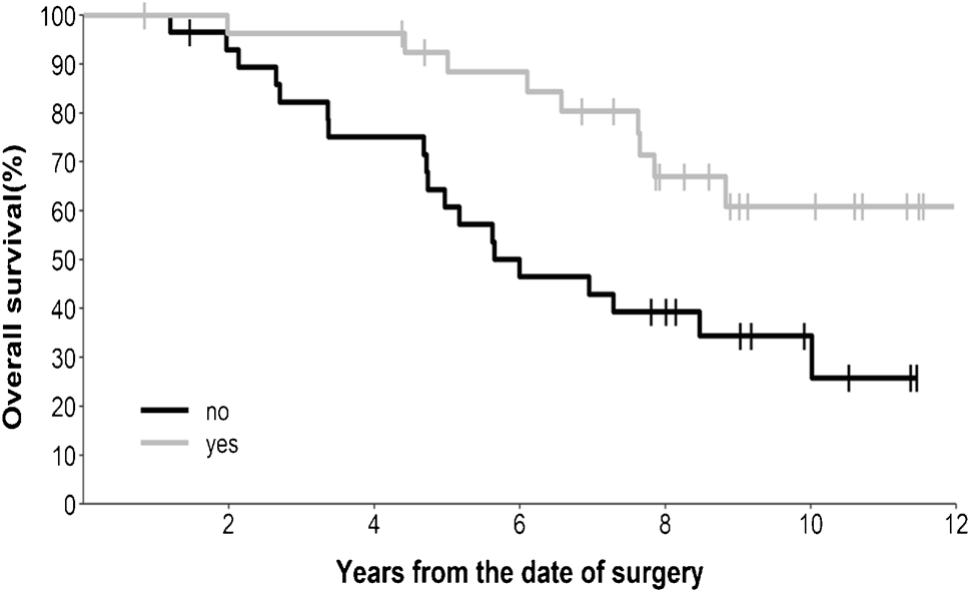
Kaplan-Meier survival curve showing the difference in patient survival depending on the presence or absence of well-defined tumour margins

**Fig. 4:**
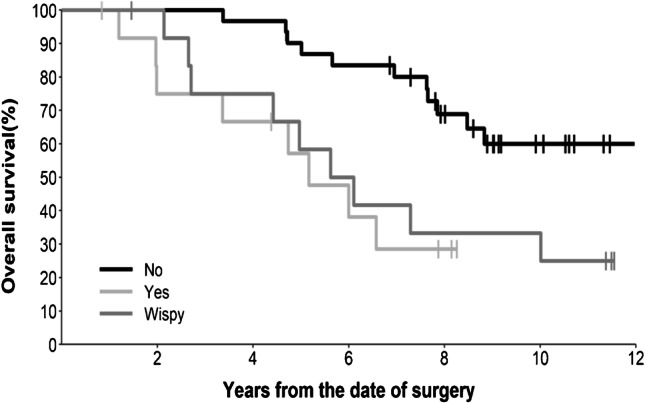
Kaplan-Meier survival curve showing the difference in patient survival based on tumour enhancement characteristics

Thirty tumours (52%) demonstrated >50% T2-FLAIR mismatch, with a further seven (12%) demonstrating 25–50% mismatch, as reported previously [24]. T2-FLAIR mismatch did not correlate with patient survival (*p*=0.977). Well-defined margins were noted in half (29/58) of the tumours and this feature was associated with longer survival (HR 0.36, 95% CI 0.16–0.79, *p*=0.008), as shown in Fig. 3. These patients did not yet reach median survival at the time of analysis, compared to a median survival of 6.0 years for patients with ill-defined tumours. In contrast, enhancement predicted shorter survival (*p*=0.004), as illustrated in Fig. 4. Fourteen tumours (24%) demonstrated solid enhancement (median survival 5.2 years; HR 3.86, 95% CI 1.52–9.82), while a further 13 (22%) demonstrated wispy enhancement (median survival 5.9 years; HR 2.92, 95% CI 1.20–7.10). This compared to a median survival of 7.8 years for tumours without enhancement. Six tumours (10%) had evidence of necrosis on MRI; a trend to short survival (HR 2.34) did not reach statistical significance (95% CI 0.69–7.92, *p*=0.159). The univariate survival results for the MRI features are summarised in Table 1.

**Table 1:** Univariate results correlating MRI features with patient survival.

	HR (95% CI)	*p* value
T2-FLAIR-mismatch		
>50%	0.92 (0.41––2.08)	*p*=0.977
25–50%	0.91 (0.28–2.90)	
<25%	ref	
Well-defined margins		
Yes	0.36 (0.16–0.79)	*p*=0.008
No	ref	
Enhancement		
Solid	3.86 (1.52–9.82)	*p*=0.004
Wispy	2.92 (1.20–7.10)	
No	ref	
Central necrosis		
Yes	2.34 (0.69–7.92)	*p*=0.159
No	ref	

*HR* hazard ratio, *CI* confidence interval, *ref* reference

The following six features were included in the multivariate Cox regression based on the univariate results: age, sex, histological grade, ECOG performance status (ECOG 0 vs ECOG 1–3), well-defined margins and enhancement. Solid enhancement remained a poor prognostic feature (HR 3.84, 95% CI 1.34–11.77, *p*=0.013), but wispy enhancement did not (HR 1.85, 95% CI 0.63–5.41, *p*=0.261). Well-defined tumour margins remained a favourable prognostic feature (HR 0.29, 95% CI 0.10–0.83, *p*=0.021). Patient age, sex and histological grade were not significantly associated with patient survival on multivariate analysis (*p*=0.843, *p*=0.286 and *p*=0.102, respectively).

None of the MRI features assessed were associated with CDKN2A/B status, with a similar distribution of MRI features in the groups with and without homozygous deletion of CDKN2A and/or CDKN2B. Importantly, four of the eight tumours with CDKN2A/B homozygous deletion were well-defined, four demonstrated ≥25% T2-FLAIR mismatch, only one demonstrated solid enhancement (with two further tumours demonstrating wispy enhancement), and none had MRI evidence of necrosis. There was also no correlation between MRI features and IDH mutation type. Of specific note, ≥25% T2-FLAIR mismatch was identified in seven of the eight tumours (87.5%) with a non-canonical IDH mutation, in additional to 30 of the 50 tumours (60%) with an R132H-IDH1 mutation, with no significant difference in the frequency (*p*=0.299). The above results correlating MRI features with CDKN2A/B status and the type of IDH mutation are summarised in Table 2.

**Table 2:** Associations between MRI features and CDKN2A/B status and IDH mutation type

	CDKN2A/B homozygous deletion			IDH mutation type		
	Yes	No	*p*-value	IDH1-R132H	Non-canonical	*p*-value
Total	8 (16%)	42 (84%)		50 (86.2%)	8 (13.8%)	
T2-FLAIR-mismatch						
>50%	3 (37.5%)	22 (52.4%)	*p*=0.743	24 (48%)	6 (75%)	*p*=0.299
25–50%	1 (12.5%)	4 (9.5%)		6 (12%)	1 (12.5%)	
<25%	4 (50%)	16 (38.1%)		20 (40%)	1 (12.5%)	
Well-defined margins						
Yes	4 (50%)	19 (45.2%)	*p*=1	24 (48%)	5 (62.5%)	*p*=0.703
No	4 (50%)	23 (54.8%)		26 (52%)	3 (37.5%)	
Enhancement						
Solid	1 (12.5%)	13 (31.0%)	*p*=0.566	12 (24%)	2 (25%)	*p*=0.975
Wispy	2 (25%)	8 (19.0%)		11 (22%)	2 (25%)	
No	5 (62.5%)	21 (50%)		27 (54%)	4 (50%)	
Central necrosis						
Yes	0 (0%)	6 (14.3%)	*p*=0.585	5 (10%)	1 (12.5%)	*p*=1
No	8 (100%)	36 (85.7%)		45 (90%)	7 (87.5%)	

## Discussion

The lack of an association between conventional MRI features and CDKN2A/B status in our cohort confirms that definitive molecular testing remains important. Given the very recent addition of CDKN2A/B homozygous deletion to the diagnostic paradigm, there remains great scope for further research in this area and associations may be found using advanced MRI techniques or artificial intelligence (AI) [27]. On the other hand, it is perhaps not surprising that we were unable to predict CDKN2A/B homozygous deletion using conventional MRI features. Histological grading and MRI assessment both assess the current status of the tumour, while CDKN2A/B homozygous deletion instead indicates a likelihood of more rapid progression down the track.

Patients with the same type and histological grade of glioma can have substantially different outcomes. This variability is only partly accounted for by new additions in WHO 2021 such as the presence of CDKN2A/B homozygous deletion for IDH-mutant astrocytomas and additional molecular features of glioblastoma for IDH-wildtype gliomas [2]. Our findings build on earlier research, with the important addition of molecular assessment according to WHO 2021, in particular the assessment of non-canonical IDH mutations and CDKN2A/B status [2]. For example, previous research using data from The Cancer Genome Atlas and The Cancer Imaging Archive produced similar results, namely that a lack of enhancement predicted longer progression-free survival in lower grade gliomas after accounting for IDH1 and 1p/19q status, while a smooth non-enhancing margin correlated with better progression-free and overall survival [28]. This cohort assessed 84 tumours across the three grade 2–3 glioma genotypes according to WHO 2016 and did not have data on IDH2 status [1]. In contrast, our 58 IDH-mutant astrocytomas were obtained from a larger cohort of 119 histological grade 2–3 gliomas [24], and we have solely assessed this genotype, rather than just correcting for genotype. Importantly, our findings provide reassurance that the correlation between imaging features and survival is not simply related to CDKN2A/B status. Indeed, conventional MRI features provided more prognostic information than CDKN2A/B status in our cohort, and more comprehensive imaging assessment would be expected to provide even more information, though further validation in larger cohorts is required. Of specific note, we have confirmed well-defined tumour margins as a favourable prognostic feature, which does not have an equivalent in WHO 2021 [2]. Therefore, despite the improvements in the WHO 2021 [2], MRI features continue to be important prognostically. MRI is complementary to clinical parameters (such as age and sex), histological features (in particular, histological grade) and molecular features, and the combination of all parameters will allow optimal prognostication.

Our findings highlight the strength of the inherently multi-parametric nature of MRI. Different MRI features, obtained as part of a single examination, provide different pieces of information. The T2-FLAIR mismatch sign is arguably the most robust radiogenomic feature [6], but provided no additional prognostic information once the diagnosis of an IDH-mutant astrocytoma has been established. In contrast, well-defined tumour margins seem to have little value for genotype prediction once a correlation with T2-FLAIR mismatch has been accounted for [24], but provided additional prognostic information within this tumour type. This may be particularly important when supratotal resection [29] is being considered. A trend towards earlier and more extensive surgical resection [30-32] and improving access to genetic sequencing methods arguably decreases the value of radiogenomics per se. However, this does not spell an end to the field, but rather an evolution [27]. Furthermore, the reluctance to adopt a “watch and wait” approach partly relates to the possibility of adverse molecular features, and such concerns may be allayed by ongoing improvements in radiogenomics.

Our finding of similar MRI appearances in tumours with both IDH1-R132H and non-canonical IDH mutations demonstrates the ability of radiogenomics to provide additional insights into tumour biology, showing that a variety of related genetic mutations result in a common imaging phenotype. This finding also reinforces that a glioma demonstrating T2-FLAIR mismatch should be considered an IDH-mutant astrocytoma even in the context of negative IDH1-R132H immunohistochemistry, with a non-canonical IDH mutation being likely. Our findings, combined with the broader literature, are sufficiently compelling that sequencing may not be required to confirm genotype, which is particularly important in centres where sequencing is difficult to obtain.

Imaging was performed on a variety of scanners, as well as incorporated from external institutions, and the radiologist readers were from different institutions. These factors provide reassurance that our findings would be relevant more broadly. The retrospective nature of our study is a limitation, as it is for the majority of this field. We acknowledge the relatively small size of our cohort, in particular tumours with CDKN2A/B homozygous deletion, related to IDH-mutant astrocytomas comprising a minority of all brain gliomas. This is compounded by CDKN2A/B homozygous deletion being known to occur in only a minority of IDH-mutant astrocytomas — about 0–12% of histological grade 2 tumours and 6–20% of grade 3 [19, 20, 33, 34]. The frequency of CDKN2A/B homozygous deletion in our cohort lies towards the upper limit of the reported range. Due to the need for a long period of follow-up, the quality of the MRIs included our study was generally lower than would be expected today, with advanced sequences now being used more commonly. Thus, modern MRI should allow for even better results.

Similarly, the management of IDH-mutant astrocytomas has inevitably evolved since the patient inclusion period. For example, there is growing interest in extending resection beyond the contrast-enhancing tumour component to the non-enhancing tumour component [30, 32]. At the time of our cohort’s diagnosis, immediate post-operative imaging for lower grade and/or non-enhancing tumours was not routine (as opposed to enhancing tumours); thus, it was not feasible to include extent of resection in our multivariate analysis, notwithstanding the challenges in accurately distinguishing between non-enhancing tumour and oedema [32]. Furthermore, extent of resection could not be considered independent of the imaging features assessed; in particular, a well-defined tumour will inherently be more amenable to more extensive resection of the non-enhancing tumour component [32]. Indeed, some of the improved prognosis related to more extensive resection of non-enhancing tumour component could simply relate to more favourable tumour morphology [32]. Equally, chemotherapy and/or radiotherapy were not administered routinely at that time and generally would have been prompted by concerning imaging features or evidence of tumour progression, thus could not be considered independent variables. We note the possibility of under-grading due to sampling error in some of our cohort, related to the inherent heterogeneity of gliomas [25], most intuitively for tumours demonstrating MRI evidence of necrosis (given that no necrosis was identified on histology). The areas of necrosis were generally small — smaller than is often seen in grade 4 gliomas. MRI evidence of necrosis is not included as a criterion for a grade 4 tumour according to the WHO classification [2] and at most can suggest a review of the available histological sample or potentially repeat sampling if it were to alter patient management [25]. WHO 2021 overcomes some of the limitations of sampling error in IDH-wildtype gliomas through the addition of molecular criteria [2], but this is less the case for IDH-mutant gliomas; thus, imaging features continue to provide prognostic value in the molecular era.

## Conclusion

Conventional MRI features did not predict CDKN2A/B homozygous deletion, but provided additional positive and negative prognostic information which was associated more strongly with prognosis than CDKN2A/B status. Therefore, similar approaches to those used to predict molecular status can provide additional information which complements the integrated histologic diagnosis. This will allow the most comprehensive overall assessment of the patient’s tumour and prognosis, as well as providing insights into the underlying tumour biology, facilitating optimal clinical management.

## Data Availability

Not applicable.

## Abbreviations

WHO: World Health Organization
IDH: Isocitrate dehydrogenase
NGS: Next-generation sequencing
FLAIR: Fluid-attenuated inversion recovery
HR: Hazard ratio
CI: Confidence interval.
